# The role of free-living amoebae in the persistence of viruses in the era of severe acute respiratory syndrome 2, should we be concerned?

**DOI:** 10.1590/0037-8682-0045-2022

**Published:** 2022-06-06

**Authors:** Beni Jequicene Mussengue Chaúque, Marilise Brittes Rott

**Affiliations:** 1 Universidade Federal do Rio Grande do Sul, Instituto de Ciências Básicas da Saúde, Departamento de Microbiologia, Imunologia e Parasitologia, Porto Alegre, RS, Brasil.; 2 Rovuma University, Department of Science, Technology, Engineering and Mathematics, Niassa Branch, Mozambique.

**Dear Editor:**

Since the beginning of the coronavirus disease 2019 (COVID-19) pandemic, humanity has experienced unprecedented and devastating consequences. Likewise, an outstanding effort has been directed to combat the virus while better understanding its biology, pathogenicity, and dynamics. Unfortunately, despite efforts to end the pandemic, we may still have to live with severe acute respiratory syndrome coronavirus 2 (SARS-Cov-2) for a long time. Although the persistence of SARS-Cov in vertebrate hosts, including bats and pangolins, is becoming increasingly clear, the role of free-living amoebae (FLA) in environmental persistence, dispersal, and perhaps the emergence of new variants needs attention. The present letter aims to shed light on the role of FLA in the environmental persistence of viruses, including SARS-Cov-2, and the possible impacts that may arise from this.

FLA are protozoa that live normally and feed freely in the environment. They are cosmopolitan, ubiquitous, and isolated from virtually all-natural environmental matrices, including plants, soil, and water. FLA have also been isolated from many anthropogenic environments, such as water from various water sources, including wells, drinking water treatment and distribution systems, pools, and sewage^1^ ^,^ ^2^. FLA isolation was also performed on various surfaces in the hospital environment, including air-conditioned dust. Some FLA can be opportunistic or pathogenic and can cause skin infections (mainly in immunocompromised individuals), keratitis, and granulomatous amoebic encephalitis (also in immunocompetent cases).

On the other hand, it has been reported that the genome, viable, and infectious viral particles of SARS-Cov-2, are present in the stools of symptomatic or asymptomatic infected persons, including those with negative results for nasopharyngeal swab tests^3^. In addition, SARS-Cov-2 has been detected in different body fluids, including urine from infected individuals. Consequently, SARS-Cov-2 was also found in sewage and in different bodies of freshwater, such as rivers that receive sewage and groundwater reservoirs^4^.

In the environment, FLA feed on particulate organic material or prey on various microorganisms, such as protozoa, fungi, bacteria, and viruses. Some of these microorganisms escape the intracellular digestion route, survive, and multiply within the FLA (in many cases, leave and recolonize the extra-amoebic environment), which is why they are called amoeba-resistant microorganisms (ARM)^5^. ARM is phylogenetically diverse and includes the main taxa with microbial representatives, including bacteria, fungi, and viruses.

Many viruses have been described as ARM, including Faustovirus, Lausannevirus, Mimivirus, Pandoravirus, Pithovirus, Yaravirus, Coxsackievirus, Adenovirus, and Human Norovirus Surrogates^6^ ^,^ ^7^. Within the FLA, viruses harbor, multiply, and modulate the internal environment of the amoebic host, affecting both its replication and spread to new hosts, as shown by Mimivirus, Marseillevirus, Tupanvirus, and Faustovirus^7^. In addition, FLA become a field of interactions between the different microorganisms it hosts, making amoebae a melting pot of genetic exchange where gene recombination is frequent^7^. The presence of ARM within the amoeba or the interaction of FLA with ARM can result in the acquisition, expression, or repression of virulence by FLA or ARM. The vast majority of microorganisms that express the ability to become ARM are pathogenic or opportunistic, Including for humans, such as Mimivirus, respiratory syncytial virus (RSV), Enterovirus, and Norovirus^6^ ^-^ ^8^.

Within the FLA, ARM is protected from environmental aggressions, including the biocidal properties of disinfectants, such as human adenovirus type 5 (HAdV 5) internalized in *Acanthamoeba polyphaga* proved to be resistant to chemical disinfectants, including high doses of sodium hypochlorite (5 mg/L)^9^. Likewise, noroviruses internalized by *Vermamoeba vermiformis*, *A. polyphaga*, and *Willaertia magna* remained stable and infectious even after exposure to lethal ultraviolet C (UVC) radiation^10^. Furthermore, FLA allow the persistence of ARM even when environmental conditions are considerably inhospitable because amoebae trophozoites turn into cysts when conditions become unfavorable. Cysts are structures of resistance to FLA, with double walls consisting essentially of cellulose and proteins. They are highly resistant to a multitude of unfavorable conditions, including extremely acidic media (pH 2), thermal shock, freezing, long-term desiccation (> 20 years), chlorine, UV, and gamma radiation. Viruses have been reported to remain viable in cysts, and infectious viral particles can be recovered from excised trophozoites^11^.

Recently, it has been demonstrated that infectious viral particles of the SARS-Cov-2 surrogate (Phi6 phage) can be recovered from infected FLA (*V. vermiformis*), including infected cysts stored for up to 20 days^12^. 

**FIGURE 1:**
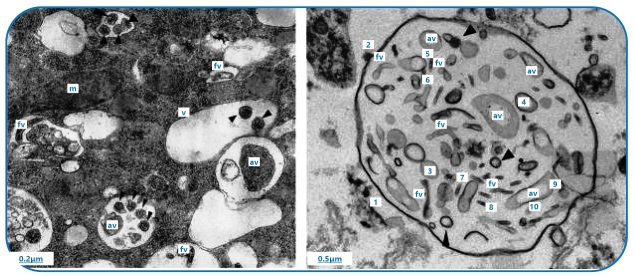
Transmission electron microscopy images showing respiratory syncytial virus (RSV) particles within *Willaertia magna* food vacuoles (left) and extracellular vesicles filled with polymorphic RSV virions (right). (m) mitochondria. (v) vacuoles. (black arrowheads) spherical virions. (av) asymmetric virions. (fv) filamentous virions^8^, with permission: https://creativecommons.org/licenses/by/4.0/.

Taking into account the previous discussion and highlighting that FLA are ubiquitous in anthropogenic and natural environmental matrices and are highly abundant in sewage, combined with the presence of SARS-Cov-2 in sewage and other contaminated water sources, it is probable that the chances of interaction of these two microorganisms in the environment are high. Notably, high titers (10^6^-10^11^) of SARS-Cov-2 have been reported in human excrement. Furthermore, although SARS-Cov-2 is considered sensitive to sewage conditions, a relatively long time (4.3-6 days) maintaining infectivity in this medium has been reported^1^ ^,^ ^2^. In addition, to FLA live and be abundant in the sewer, a relatively short time (one hour) of interaction between FLA and viruses has been reported to be sufficient to internalize a considerable number of viral particles (4 × 10^2^), as shown for human norovirus (HuNoV) surrogates (*Murine norovirus* type 1 and *Feline calicivirus*)^11^. FLA usually disperse internalized viruses by releasing respirable-sized vesicles (2-3 µm) filled with viral particles (Figure 1), as shown for RSV and Coxsackievirus B5^12^. This may increase the resistance, viability time, and range of dispersion of SARS-Cov-2 in the environment since the vesicles provide protection against biocidal factors until they reach a new host or less unfavorable environments^8^ ^,^ ^12^. The public health concern raised by this possibility is more significant for rural areas of developing countries, where sanitation is critical, and people's exposure to contaminated water is greater. Furthermore, the interaction of SARS-Cov-2 with other ARM in the intracellular environment of FLA may favor gene recombination, which can result in the regressive or progressive evolution of the virus.

It has been speculated that FLA could serve as a model to study the pathogenesis of SARS-Cov-2. This speculation was based on the characteristics of *Acanthamoeba* (e.g., molecular motility, biochemical physiology, phagocytosis, and interactions with pathogens) that have notable parallels with macrophages. Furthermore, it has been reported that *Naegleria fowleri* can express primitive forms of angiotensin-converting enzyme 2 (ACE2) and transmembrane serine protease 2 (TMPRSS2) proteins, and *Acanthamoeba* spp. and *N. fowleri* can code for furin, which are proteins that have been reported as binding sites for SARS-Cov-2 in human cells.

The results of studies that aim to evaluate the *in situ* and *in vitro* interaction of SARS-Cov-2 with FLA are desirable and of great value to support the fight against the ongoing pandemic and future ones.
